# Size and Shape of Primary (Bio)Polyelectrolyte Complexes Chitosan/Gelatin: Study Using Small-Angle X-Ray Scattering from Synchrotron Radiation

**DOI:** 10.3390/polym17091236

**Published:** 2025-04-30

**Authors:** Aleksandr Podshivalov, Mikhail Litvinov, Aleksandr Kashurin, Ksenia Danilova

**Affiliations:** Center for Chemical Engineering, ITMO University, Kronverkskiy Prospekt, 49, 197101 Saint-Petersburg, Russia; mikhail.litvinov.1996@mail.ru (M.L.); pkashurin@mail.ru (A.K.); k.danilova@itmo.ru (K.D.)

**Keywords:** chitosan, gelatin, polyelectrolyte complex, SAXS, synchrotron radiation

## Abstract

In this work, using small-angle X-ray scattering from synchrotron radiation, the macromolecular structure of chitosan and gelatin polyelectrolytes and their mixtures at various pH values and ratios was studied to determine the size and shape of primary supramolecular (bio)PEC. Analysis of the scattering profiles of the initial solutions of chitosan and gelatin with the building of the pair distance function showed the formation of single-modal distributions with a maximum molecular size of 46 and 32.2 nm, respectively. Ab initio reconstruction of the macromolecule’s shape showed the formation of objects shaped like an oblate spheroid. In mixtures of chitosan and gelatin at a pH below the isoelectric point, it was found that the scattering structures correspond to the initial biopolymers. However, it is observed that values of the aspect ratio at a ratio above 1:10 gradually increase, which indicates a slight elongation of the average particle and indirectly indicates the formation of dissipative structures of (bio)PEC. In mixtures at a pH above the isoelectric point, it was shown that at ratios above 1:5, the formation of primary supramolecular complexes is observed, which is accompanied by an increase in zero-scattering intensity by about three times, maximum molecular size by two to two-and-a-half times relative to the initial polymers, and the formation of elongated structures corresponding to the cylinder (swollen spiral). It may be a consequence of the increased efficiency of the polyelectrolyte associative interaction between chitosan and gelatin.

## 1. Introduction

Recently, much attention has been paid by researchers in the field of drug delivery systems and gene carriers to the investigations of self-assembly of interpolyelectrolyte complexes (polyelectrolyte coacervates) [1,2,3,4]. These complexes are formed because of the electrostatic interactions of oppositely charged polyelectrolytes. They can spontaneously form various complex structures: spherical micelles, complex aggregates (coacervates), and solid-phase supramolecular structures [5,6]. The analysis of the existing mechanisms of coacervation can be combined into the following stages [7,8]: (1) pairing of oppositely charged ions with the formation of intermediate supramolecular assemblies (soluble PEC); (2) rearrangement of soluble complexes into the coacervate phase due to non-electrostatic interactions; and (3) with a non-stoichiometric charge balance, excess chains of polyanions or polycations can attach to the surface of the complex, causing repulsive interactions, which leads to colloidal stability. With a stoichiometric charge balance, subsequent self-assembly occurs, with the formation of a solid-phase supramolecular structure.

Numerous works [9,10,11,12,13] studying the self-assembly of PEC structures mainly concerned the stages of formation of the coacervate phase and investigated the conditions for obtaining these structures (concentration, ratio, pH, and ionic strength), as well as the effect of these conditions on the size, structure, polymorphism, and stability of coacervates. However, at present, not enough work is devoted to the study of the primary stage of PEC formation, which is mainly due to the dissipativity of the primary supramolecular assemblies formed and the high constant rate of binding of ion pairs. At the same time, existing light scattering methods allow us to estimate only the evolution of the overall size of submicron particles, but at the same time they do not provide any information about the structure of aggregates and provide little information about the details of the structure of nanoparticles. Currently, synchrotron or neutron radiation methods are used to study data on the size, shape, and internal structure of primary complexes at the nanoparticle level [6,14,15,16,17,18,19]. For example, in the work using small-angle X-ray scattering with time resolution [6], the kinetics of coacervation of sodium polyacrylate and polyallylamine hydrochloride were studied and it was established that at the initial stage, the formation of almost neutral particles of primary coacervates occurs, which subsequently assemble into large coacervates with evolution over time comparable to the rate of Brownian coagulation in the presence of salt. In another work [16] using neutron and X-ray scattering, the coacervation of poly(acrylic acid) and poly(N,N-dimethylaminoethylmethacrylate) was studied, and the formation of an ideal Gaussian conformation of the polymer chain was discovered in complex coacervates forming a strongly intertwined network with a cell size much smaller than the radius of gyration of polymers. These works are related to the mechanisms of formation of PEC based on homopolymer polyelectrolytes. However, PEC formed from two-block copolyelectrolytes with charged and neutral blocks is of great interest. In such systems, the stabilization of coacervates strongly depends on the aggregation of neutral blocks. So, for example, in the works [14,17], the kinetic pathways of the formation of micellar-like PEC formed from anionic and cationic neutral block copolymers were studied and it was found that metastable cylindrical aggregates are formed at the first stage of complexation. Further, they regroup when the charge is balanced into stable micellar-like aggregates. At the same time, the rate of regrouping strongly depends on the MW of the neutral block. However, despite the great scientific foundation, the works described above mainly concerned PECs based on synthetic polyelectrolytes.

Among biopolymers, special attention should be paid to chitosan, which is a natural copolymer consisting of polycation units of glucosamine and neutral units of acetylglucosamine, and its complexes with polyampholytic protein hydrolysates containing both neutral, anionic, and cationic units. Among such systems, chitosan-gelatin (bio)PEC is of interest because of their availability for creating drug delivery systems. The formation of (bio)PEC in the chitosan-gelatin system has been extensively studied in the literature, but most studies have focused exclusively on the macroscopic aspects of coacervation. In numerous works [20,21,22,23,24,25,26,27,28,29], the process of coacervation at a pH above the isoelectric point of gelatin has been described in detail. For example, it was shown in work [24] that the formation of a coacervate phase between chitosan and gelatin type B is possible in the pH range of 5.5–6.5 with a minimum polymer ratio of 1:10. Moreover, at a ratio of 1:20 and pH = 6, coacervates with a zeta potential close to 0 and a diameter of ~30 μm are formed. In another work [25], it was shown that the formation of the coacervate phase in a mixture of chitosan and gelatin type B solutions at a ratio of 1:2 is accompanied by a significant increase in the turbidity of the solution, with maximum values at pH = 5.5. It is also shown that the formation of the coacervate phase under these conditions leads to an increase in the particle size of the dispersed phase by more than five times compared with mixtures at pH = 3. In our previous work [29], we also studied the formation and structure of the coacervate phase of (bio)PEC in mixtures of dilute chitosan and gelatin type B solutions. It was shown that the formation of stable dispersions of coacervates with a size ranging from 3 to 4.5 μm is observed in the pH range from 5 to 6.4 and a polymer ratio above 1:5. Additionally, using the dynamic light scattering method, it was shown that the coacervate phase of the (bio)PEC consists of submicron particles of primary complexes and micron-sized coacervates.

An analysis of the literature in the field of chitosan-gelatin (bio)PEC shows that the main works focus on the effect of the pH of the medium and composition on the formation of dispersions of complex particles and their morphology, studied mainly by optical microscopy, turbidimetry, and dynamic light scattering. However, the shape and size of the primary (bio)PEC structures have not been studied in detail. In this work, we solve this fundamental problem by applying synchrotron SAXS research with the analysis of pair distance distribution functions (PDDF) to systematically study the evolution of the sizes of primary structures of (bio)PEC depending on the composition and pH of the medium, the mechanisms of their formation at the molecular level, and to study the shape of primary structures of (bio)PEC. This approach will make it possible for the first time to obtain detailed information about the nanostructure of the forming complexes, which is not available for traditional optical methods.

## 2. Materials and Methods

### 2.1. Materials

We used gelatin type B produced by Sigma-Aldrich Corporation (St. Louis, MO, USA) (CAS 9000-70-8) with a viscous average molecular weight of *M_v_* = 63.1 ± 8.7 kDa and an isoelectric point of pI = 4.7 ± 0.3, which was determined by viscometric and turbidimetric methods in previous work [29]; chitosan produced by CJSC “BioProgress” (Losino-Petrovsky, Russia) with *M_v_* = 206 ± 1.5 kDa, protonation constant *pK*_0_ = 6.35, and degree of deacetylation of DD = 83.1% [30]; glacial acetic acid, sodium hydroxide produced by JSC “LenReactiv” (Saint Petersburg, Russia) and deionized water.

### 2.2. Preparation of Chitosan/Gelatin (Bio)Polyelectrolyte Complex Dispersions

To obtain a dispersion of (bio)PEC between chitosan and gelatin 0.5 M acetic acid solutions of chitosan with *C* = 1 mg/mL and gelatin, with *C* = 5 mg/mL were prepared over 24 h at a temperature of 35 °C with constant stirring using a magnetic stirrer. After preparation, the polymer solutions were centrifuged using an Eppendorf MiniSpin Plus centrifuge, Eppendorf AG (Hamburg, Germany) at 10,000 rpm for 10 min. Further, the obtained solutions were mixed in the following ratios of 1:1, 1:5, 1:10, 1:15, and 1:20 by weight over 1 h at a temperature of 35 °C with constant stirring. After that, the obtained solutions were adjusted to pH = 3 by titration with 0.5 M acetic acid and to pH = 5.5 with 1 M and 2 M of NaOH solutions. These pH = 5.5 values were chosen because it was previously established [29] that at a pH above the isoelectric point, effective formation of scattering particles (bio)PEC occurs, which manifests itself in significant turbidity of the solution.

### 2.3. Small-Angle X-Ray Scattering of Synchrotron Radiation

Data on the X-ray scattering of synchrotron radiation were obtained on the beamline of the BioMUR station of the synchrotron radiation source (National Research Center “Kurchatov Institute”, Moscow, Russia) [31]. The BioMUR experimental station consists of optical and experimental parts. In the optical part, a beam is formed in the geometry of the three-slit collimation, which is focused using a Si (111) monochromator mounted at a Bragg angle of 13.5° and equipped with a stepper motor for bending the crystal and focusing the beam in the horizontal direction. The beam energy after the monochromator crystal is 8.58 keV (*λ* = 0.1445 nm). SAXS data were recorded using a two-dimensional pixel detector, Pilatus-3 1M (DECTRIS, Baden-Daettwil, Switzerland), with a pixel size of 172 μm and a recording area of 168 × 179 mm. The beam size on the detector was approximately 0.5 × 0.35 mm. To measure the intensity of small-angle X-ray scattering, a sample of the solution was placed in a quartz capillary with a volume of 100–200 µL with a wall thickness of no more than 0.01 mm. We used capillary QGCT 2.0 mm with a diameter of 2 mm and a length of 80 mm, manufactured by Capillary Tube Supplies Ltd., Cornwall, UK. The distance between the sample and the detector was 700 mm, which corresponds to the angular range of the scattering vector *q* = 0.1–4.0 nm^−1^, calculated according to Equation (1):

(1) $$q=\frac{4\pi \sin\theta }{\lambda },$$

where 2*θ* is the scattering angle, and *λ* is the wavelength of the X-ray radiation. The sample was exposed to monochromatic X-ray radiation for at least 600 s. The transition to the scattering vector modulus scale was performed by measuring the diffraction standard for AgBh. This transformation and the averaging of the two-dimensional diffraction scattering pattern were performed using the Fit2D software v. 17.006 (Andrew Peter Hammersley, European Synchrotron Radiation Facility, Grenoble, France) [32]. The built-in software module “PRIMUS” of the ATSAS software v. 3.2.1 (European Molecular Biology Laboratory, Hamburg, Germany) [33] was used to subtract the scattering contribution of the buffer solution from the scattering curves of the studied samples.

For the obtained experimental SAXS curves, the fractal dimension of the particle was determined by its scattering profile in a double logarithmic format, analyzing the power mode. In this mode, it was shown that *I(q)* follows the power form [34,35] in Equation (2):

(2) $$I\left(q\right)\propto q^{-\alpha },$$

where *α* is the exponent associated with the fractal dimension of the scattering structures. For mass fractals, it can be shown that *α* = *D_m_* and 1 *< α* < 3 in three-dimensional space. In addition, the scattering data were qualitatively evaluated using the Kratky plot (*I(q)q^2^* vs. *q*).

The obtained experimental SAXS curves were analyzed in the built-in software module “GNOM” of the ATSAS software using the indirect Fourier transform of the distribution function of paired distances *P(r)* according to Equation (3) [36]:

(3) $$P(r)=\frac{r^{2}}{{2\pi }^{2}}\int_{0}^{\infty }q^{2}I(q)\frac{sin(qr)}{qr}dq,$$

where *r* is the minimum distance between particles (atoms) in the structure of the substance. The values of the root-mean-square radius of gyration of *R_g_*^2^ particles were also determined using PDDF as Equation (4):

(4) $$R_{g}^{2}=\frac{\int_{0}^{D_{max}}r^{2}P\left(r\right)dr}{2\int_{0}^{D_{max}}P\left(r\right)dr},$$

where *D_max_* is the maximum particle size in the dispersion. Next, the value of the zero-scattering intensity (direct scattering) *I*_0_ was determined as Equation (5):

(5) $$I_{0}=4\pi \int_{0}^{D_{max}}P\left(r\right)dr,$$

### 2.4. Determination of the Molecular Weight of Biopolymers Based on SAXS Data

The molecular weight of chitosan and gelatin biopolymers was determined through the correlation volume *V_c_*, which was determined as the ratio of the zero-scattering intensity *I*_0_ to the total scattering intensity in Equation (6) [37]:

(6) $$Vc=\frac{I_{0}}{\int_{0}^{\infty }I\left(q\right)\;q^{2}\;dq},$$

*MW* was determined by the empirical *V_c_*^2^*/R_g_* ratio using Equation (7):

(7) $$MW={\left(\frac{V_{c}^{2}/R_{g}}{c}\right)}^{k},$$

where *c* and *k* are empirically determined constants based on the fitting of theoretical scattering profiles: *c* = 0.1231 and *k* = 1. The *V_c_* values for gelatin were 884 Å^2^, and the calculated values of *MW* were 58.9 kDa. The values obtained show good convergence with the results of the determination of the average viscous *MW*. Despite the use of constants for proteins, these constants have also been used to determine the *MW* of chitosan, as this method can be used for both flexible and extended macromolecules. As a result, for chitosan, *V_c_* = 2115 Å^2^ and *MW* = 213.8 kDa. The obtained values also converge with the values of the average viscous *MW*.

### 2.5. Ab Initio Modelling

Ab initio reconstruction of the shape of scattering objects was carried out using the annealing simulation algorithm implemented in DAMMIN (Dummy Atom Model Minimization) [38]. In this method, a dummy atom model is generated by filling a sphere with a diameter equal to the maximum particle size, *D_max_*. In this case, each dummy atom must be attributed either to a particle or to a solvent. Annealing modeling consists of calculating the scattering intensity from an arbitrary starting configuration of fictitious atoms, and then during the annealing process, the theoretical scattering intensity is calculated from random modifications of this configuration in order to minimize *χ*^2^ as a result, that is, to obtain the minimum discrepancy between the experimental and model scattering curves according to Equation (8):

(8) $$\chi^{2}=\frac{1}{N-1}\sum_{j}{\left[\frac{I_{exp}\left(q_{j}\right)-cI_{calc}\left(q_{j}\right)}{\sigma \left(q_{j}\right)}\right]}^{2},$$

where *N* is the number of experimental points, c is the scaling factor; *I_exp_(q_j_)* is the experimental intensity; *I_calc_(q_j_)* is the calculated intensity in the model; *σ(q_j_)* is the experimental intensity error. The stability of the reconstructed models was tested by repeated modeling. Averaging of 10 models was carried out using DAMAVER [39], which overlays all models and defines a common shell containing all models.

To reduce the effective number of free parameters in the model, the method searches for configuration X minimizing (9) [40]:

(9) $${f\left(X\right)=\chi }^{2}+\alpha P(x),$$

where *P(x)* is the looseness penalty, and *α* is the weight of the penalty. The weight of the penalty is selected to have a significant penalty contribution at the end of the minimization. A final compact and interconnected bead configuration is characterized by *P(x)* ≈ 0.01, *α* ≈ 10, and *χ^2^* ≈ 0.6–2.5. Visualization of the obtained models was carried out in PyMOL.

### 2.6. Dynamic Light Scattering

The dynamic light scattering (DLS) method was used to determine the particle size distribution in chitosan and gelatin solutions and their mixtures at pH = 5.5. The study was carried out using the particle size analyzer LB 550, Horiba (Edison, NJ, USA) using a laser beam with a wavelength of 650 nm, a power of 5 mW, and a scattering angle of 177°. Each sample was measured at least 5 times. The measurements were carried out at a temperature of 25 °C. Before the measurement, all solutions were additionally diluted with distilled water by a factor of 10.

## 3. Results

### 3.1. Analysis of Conformation and Macromolecular Sizes of Individual Chitosan and Gelatin Solutions Using SAXS

**Figure 1:**
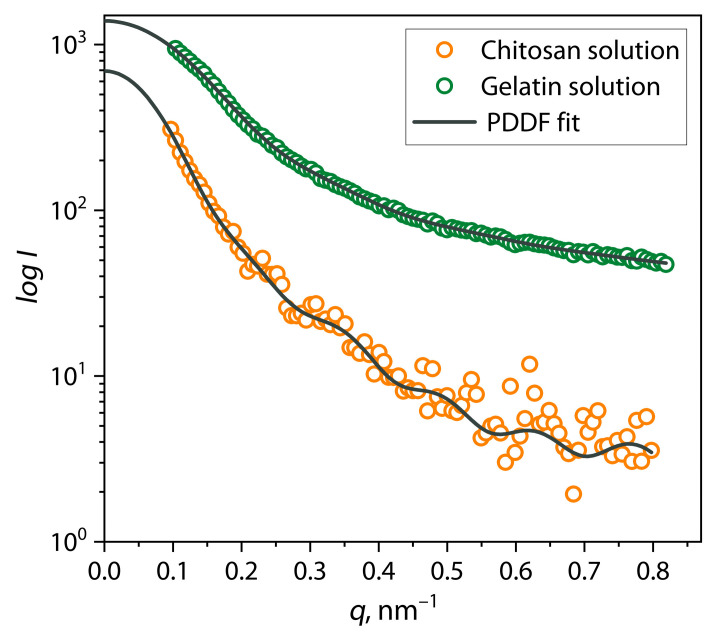
SAXS patterns of individual chitosan and gelatin solutions at pH = 5.5.

Before studying the structure of PECs and the mechanism of their formation, the sizes and the conformations of gelatin and chitosan macromolecules in a buffer solution were first studied. For this purpose, analyses of the individual diluted solutions of gelatin with a concentration of *C* = 1 mg/mL and chitosan with *C* = 5 mg/mL at pH = 3 and 5.5 were carried out using small-angle X-ray scattering (SAXS) with synchrotron radiation. It is important to note that during the measurements of solution samples at pH = 3, low-intensity scattering patterns were observed, and they were considered inadequate; therefore, they are not presented in this work. This result may be associated with significant polyelectrolyte swelling of gelatin protein and chitosan macromolecules because of intense repulsive interaction of similarly charged amino groups in acidic media, leading to the formation of a small number of large-sized scattering centers. Thus, the scattering patterns of biopolymer solution samples at pH = 5.5, presented in Figure 1, were subjected to analysis.

The graph shows that the intensity of radiation scattering in a gelatin solution sample significantly exceeds the intensity in a chitosan solution sample. This can be due not only to the difference in polymer concentration but also to their scattering contrast. Gelatin, as a protein polymer, has a higher electron density compared to polysaccharide chitosan, which can lead to higher scattering intensity. In addition, this effect is most likely a consequence of both the low solubility and the high lability of chitosan macromolecules in the buffer solution used at pH = 5.5 due to its polycationic nature, in contrast to proteins in gelatin, which have amphoteric properties. Figure 1 shows that at high values of the scattering vector *q*, a noisy signal is observed in the scattering pattern (at wide scattering angles) of a chitosan solution sample, which is also associated with a small number of individual chitosan macromolecules compared to associates. Due to the differences in the configuration of the macromolecules of gelatin proteins and chitosan, as well as their different charge balance, under these conditions, gelatin macromolecules are able to effectively swell to form a “true” solution, while chitosan macromolecules can form labile associates, with the formation of a dispersion.

The configuration difference between chitosan and gelatin macromolecules in their dilute solutions is also reflected in a number of works. For example, in work [41], using the DLS and SAXS experiment, it was shown that gelatin solutions are characterized by the so-called collapse-swelling state transition. This transition reflects the fact that gelatin macromolecules outside the isoelectric point swell and transform from a globule into a highly swollen coil [42]. As for chitosan, its key problem is the production of “true” aqueous solutions, which is complicated by its chitinous nature. Chitosan aqueous solutions are typically dispersions containing polymeric macromolecules and labile associates (aggregates) [43,44,45]. The formation of chitosan aggregates, leading to a decrease in the intensity profile, is mainly caused by the association of *N*-acetyl-*D*-glucosamine units due to hydrophobic interactions, as well as the hydrophilic interaction of *D*-glucosamine units with the formation of hydrogen bonds [44].

**Figure 2:**
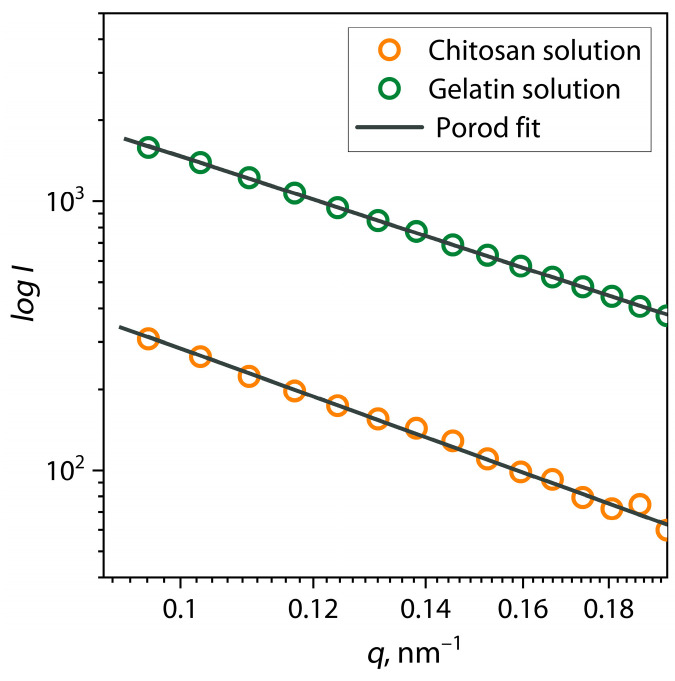
Porod approximation of SAXS patterns of individual chitosan and gelatin solutions at pH = 5.5.

For a rough analysis of the fractal dimension and shape of macromolecular coils of biopolymers in solution, using the Porod approach, linear regions of the scattering patterns in the range of *q* ≤ 0.2 were analyzed, and the value of the *α* exponent was determined (Equation (2)). A visual representation of such an analysis is presented in Figure 2.

According to this approach, the value of the *α* exponent (slope coefficient) contains information about the distribution of interatomic distances inside the molecule and allows us to estimate the fractal dimension and surface quality of scattering objects. As a result, the calculated values of the exponent were *α* = 2 for a gelatin solution and *α* = 2.26 for a chitosan solution. Such values correspond to scattering on macromolecules or supramolecular structures such as a random coil in a *Θ*-solvent [41,46,47]. The results of the analysis of scattering patterns of samples of gelatin and chitosan solutions are also presented in Table 1.

For example, in the work [47], when analyzing the scattering profiles of mixtures of solutions of the protein rezilin and polypeptide, the values of *α* equal to 2 are shown. For an aqueous gelatin solution [41], values close to 2 were also observed. In addition, such structures analyzed using synchrotron radiation correspond to theoretical and practical studies of conformational states in chitosan and gelatin, using DLS and TEM. For example, in works [43,44,48,49] using DLS, TEM, and AFM, it was shown that chitosan macromolecules can take conformations from a random coil to a rigid rod, depending on the molecular weight and degree of deacetylation. In work [49], it was shown that the conformation of chitosan aqueous solutions changes from a random coil to a rigid rod, with a decrease in pH from 3.5 to 1.55, which was explained by an increase in the chain charge. In other works [42,50], for gelatin macromolecules at a pH above pI, the main conformation is a swollen random coil. It is worth noting that the conformation of the random coil for chitosan solutions is due to the effect of charge screening in the buffer solution, which reduces its electrostatic repulsion and prevents its elongation into a rod shape. A similar effect, but to a lesser extent (due to the low content of ionogenic groups), is typical for gelatin solutions at a pH below and above the isoelectric point.

**Figure 3:**
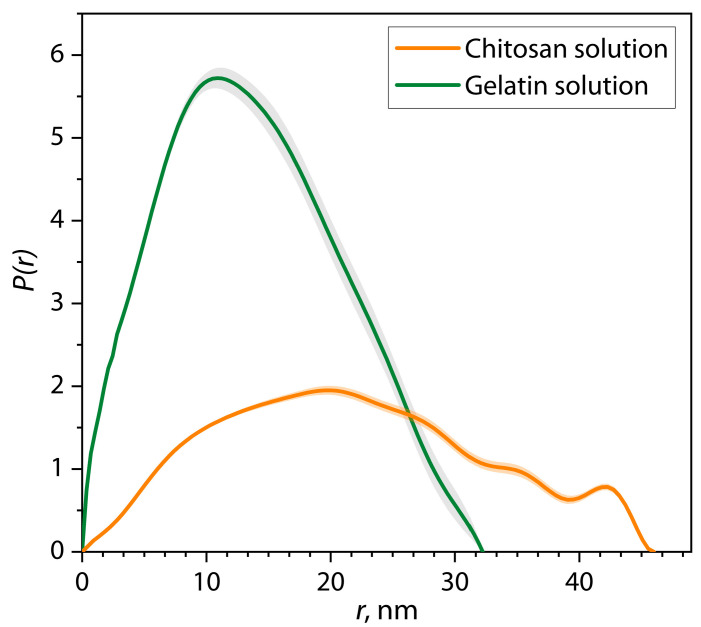
Pair-distance distributions of individual chitosan and gelatin solutions at pH = 5.5.

For a more detailed study of the equilibrium size and shape of biopolymer macromolecules under selected conditions, small-angle scattering patterns were analyzed using the pair interatomic distance distribution function (PDDF). The PDDF function is a distribution of interatomic distances (*r*), which was calculated using the inverse Fourier transform of the scattering patterns according to Equation (3), as shown in Figure 2 by dark gray lines. In addition, this approach allows the scattering patterns to be interpolated into the region of the zero value of the scattering vector *q* (the smallest angles) to determine the value of zero scattering intensity *I*_0_, which is mathematically expressed as Equation (5). The results of the analysis of scattering patterns of samples of gelatin and chitosan solutions using the PDDF function and *I*_0_ values are presented in Table 1. Figure 3 shows the pair distance distribution function for chitosan and gelatin solutions at pH = 5.5.

In general, the PDDF function demonstrates the distribution of pair interatomic distances along their length in the structure of the average scattering object. At maximum convergence of the PDDF function with the experimental scattering pattern in the sample, the limiting interval of such a distribution is the value of *D_max_*, corresponding to the maximum diameter of the averaged scattering object. Moreover, the intensity and width of the distribution provide information about the shape of the averaged scattering object.

It can be seen from the figure that the PDDF distribution for a gelatin solution is a wide, single-modal distribution and has a shape close to a normal symmetric distribution, with a maximum intensity value *P(r)* = 5.72 at *r* = 10.9 nm and a maximum diameter *D_max_* = 32.2 nm for macromolecules of protein in gelatin. The semi-transparent areas on the figure represent the uncertainty range in the PDDF function. At the same time, it is clear that the distribution for the chitosan solution demonstrates a much lower maximum intensity *P(r)* = 1.9 at *r* = 19.5 nm, with a larger width and *D_max_* = 46 nm. Compared with the curve for a gelatin solution, the graph for a chitosan solution is characterized by a wider and less intense distribution, which, as previously explained, is associated with a different scattering contrast and a lower number of scattering objects in the solution. It is noteworthy that the zero scattering intensity value *I*_0_ in the chitosan solution is reduced by about two times compared to gelatin solution.

As part of the PDDF analysis, it is also possible to establish the average value of the hydrodynamic radius *R_g_* for scattering objects using Equation (4). The calculated values were *R_g_* = 10.8 ± 0.2 nm for the gelatin solution and *R_g_* = 17.0 ± 0.1 nm for the chitosan solution. These calculations show large differences in the density of macromolecular coils of biopolymers in solution compared to a comparison of the values of their maximum diameter *D_max_*. To numerically estimate the “density” and shape of macromolecular coils of biopolymers in solution, we used the aspect ratio *AR*, calculated using Equation (10) (Table 1). *AR* values show that the macromolecules of gelatin and chitosan, despite the difference in size, have a similar ellipsoidal shape, close to spherical.

According to the literature [38], it is known that the shape of the distribution of the PDDF function also makes it possible to determine the shape of the average scattering object in the sample. PDDF distributions for solutions of gelatin and chitosan in Figure 3 have a dome-shaped form close to a symmetrical distribution, while the distribution for the gelatin solution is more intense and less wide than that for the chitosan solution. This shape corresponds to a close-to-spherical form for gelatin macromolecules and an ellipsoidal flattened form for chitosan coils, which confirms the results of calculations of the α exponent in the Porod analysis and the *AR* ratio.

**Figure 4:**
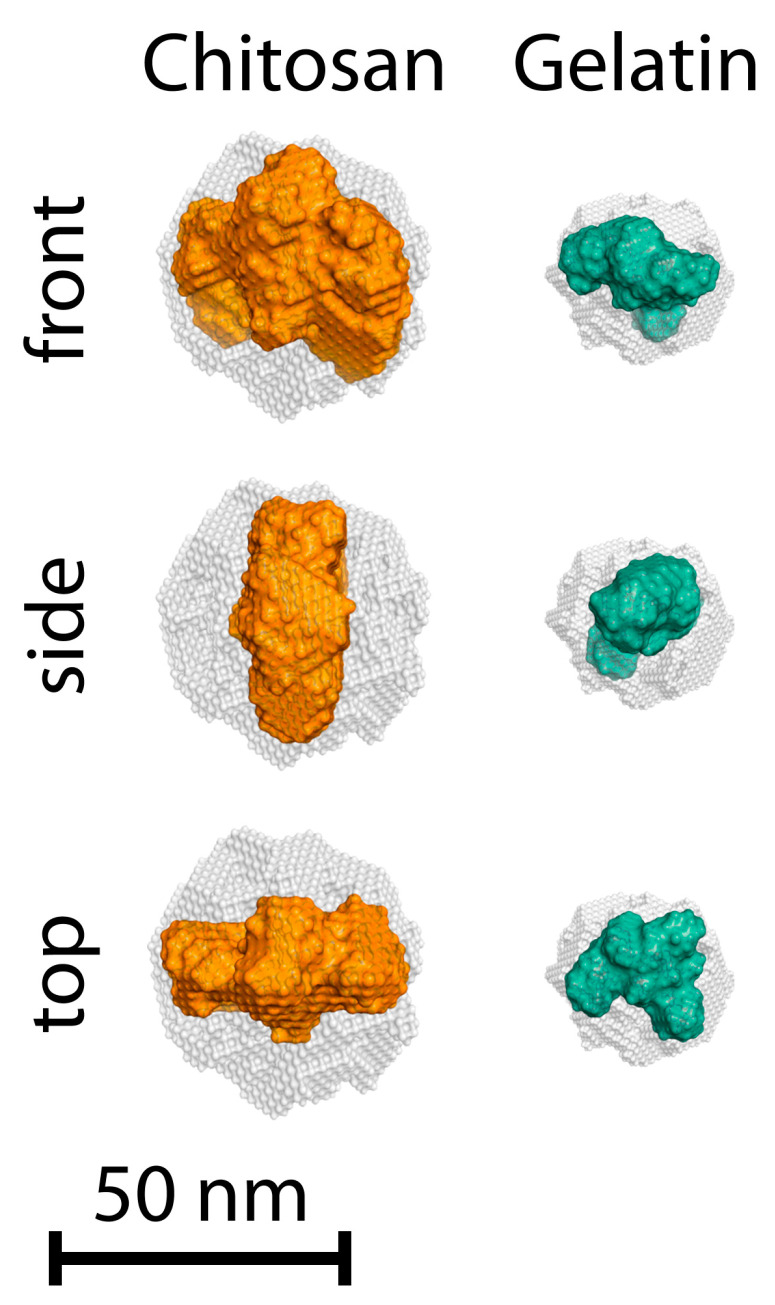
Ab initio models of chitosan and gelatin macromolecules using Equation (8).

An additional analysis of the shape of chitosan and gelatin macromolecules was carried out using ab initio reconstruction modeling. Figure 4 shows the obtained models of dummy atoms in front, side, and top projections.

The gray semitransparent spheres in the figure framing the obtained models of biopolymer macromolecules correspond to the initial random-phase distribution in the search volume, with a diameter equal to *D_max_*. It can be seen from the figure that the scattering structures correspond to their maximum molecular sizes and represent a compressed model of tightly packed dummy atoms (in color). Visually, it is noticeable that chitosan macromolecules are characterized by structures resembling an oblate spheroid, while gelatin is characterized by a more compact and less ordered shape. The modeled macromolecules of gelatin and chitosan, in their shape, correspond well to the structure characteristic of partially unfolded proteins, for which the most advantageous conformation is a random coil. The analysis of scattering profiles and the ab initio reconstruction of the shape of these proteins have been studied in detail in works [51,52,53].

### 3.2. Estimation of Self-Assembly of Primary PEC Structures Using SAXS

As described above, in aqueous solutions, the chitosan macromolecules can have a positive charge due to the protonation of open side deacetylated amino groups. Under the same conditions, gelatin polypeptide macromolecules can receive a dual charge: positive during the protonation of open side amino groups of predominantly arginine and lysine in the structure of the α-chains of polypeptides, and negative during the electrolytic dissociation of the carboxyl groups of aspartic and glutamic acids, correspondingly.

Such processes lead to significant swelling of the macromolecules of these polymers due to the strong electrostatic repulsion of similarly charged groups of macroions. Naturally, the strength of the effect of polyelectrolyte swelling of macromolecules depends on the efficiency of protonation of amino groups in the presence of a large number of protons in an acidic medium, and the efficiency of dissociation of acid groups in the presence of hydroxyl ions in an alkaline solution.

In the case when chitosan and gelatin macromolecules are simultaneously present in an acidic medium in a swollen state, the effect of their electrostatic repulsion from each other is observed due to the high positive charge density of equally charged macromolecules or polymer particles. On the contrary, when the macromolecules of chitosan and gelatin are in an alkaline environment, the protonation of amino groups in chitosan and gelatin is almost completely suppressed, but the dissociation of acidic groups in gelatin proteins is significantly enhanced. As a result of these processes, the gelatin proteins swell and repel each other to form a true solution, while the chitosan macromolecules collapse into an uncharged state and strongly associate, forming a dispersion of chitosan particles in the gelatin solution [24,29].

Despite this, there is a “window” in the pH range between the isoelectric point of gelatin proteins and the characteristic protonation constant of chitosan, pI < pH < pK_0_, in which the protonation of the amino groups of both polymers and the dissociation of the acidic groups of gelatin proteins occur simultaneously. Under such conditions, the chitosan macromolecules are weakly charged positively, and the macromolecules of gelatin proteins partially have a negative charge. These conditions lead to the activation of electrostatic attraction of chitosan and gelatin macromolecules, followed by their ionic bonding at the sites of oppositely charged functional groups and the formation of the primary structures of a (bio)polyelectrolyte complex ((bio)PEC). Such structures, as a rule, consist of several bound macromolecules in an unstable state due to an imbalance of surface charge. Moreover, these structures belong to supramolecular formations with large size and mass relative to the individual macromolecules of biopolymers in solution. The charge imbalance on the surface of the primary (bio)PEC structures leads to their further association with each other due to electrostatic attraction, with the formation of nano- or microdispersion, which is also confirmed by a significant increase in the optical density of the solution due to the intense light scattering of the mixture of chitosan and gelatin when the condition pI < pH < pK_0_ is met. However, it is clear that methods, such as turbidimetric photometry and dynamic light scattering (DLS), do not allow for accurate assessment of the size and shape of primary (bio)PEC structures.

**Figure 5:**
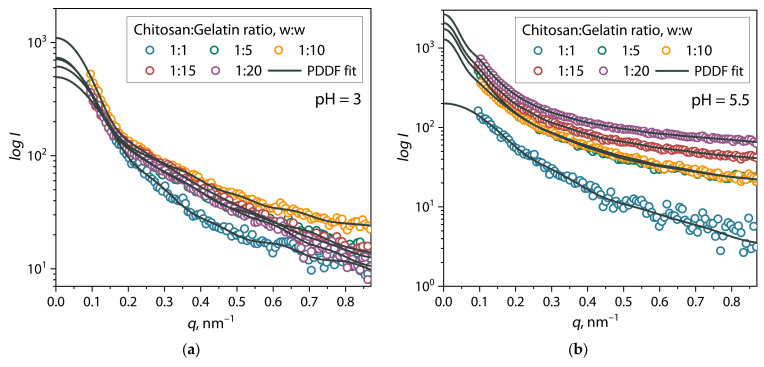
SAXS patterns of chitosan/gelatin mixtures at (**a**) pH = 3 and (**b**) pH = 5.5.

Establishing the size and shape of primary (bio)PEC structures as a function of the chitosan/gelatin ratio and pH level with higher resolution using small-angle X-ray scattering with high-intensity synchrotron radiation can provide important information for a deeper understanding of the mechanisms of the formation of these structures. For this reason, we studied the scattering patterns of SAXS with synchrotron radiation for samples of (bio)PEC dispersions at pH = 3 and 5.5 (below and above the pI value for gelatin) depending on the chitosan/gelatin mixture ratio. The SAXS scattering patterns for (bio)PEC dispersions at different biopolymer ratios are presented in Figure 5.

Scattering patterns for chitosan/gelatin mixture dispersions at pH = 3, presented in Figure 5a, show that the scattering intensity varies independently of the ratio within a certain range between the values corresponding to the initial biopolymers. A completely different picture is observed for chitosan/gelatin dispersions at pH = 5.5 (Figure 5b). It can be seen that, in general, the intensity of the scattering curves is greater for all dispersions except the 1:1 mixture compared to the curves for dispersions at pH = 3. Moreover, in this case, the effect of the influence of the chitosan/gelatin ratio in the mixture is significantly higher: with an increase in the proportion of gelatin in the mixture, the signal becomes less noisy and more intense.

Analyzing and comparing the SAXS results for chitosan/gelatin dispersions at pH = 3 and 5.5, we came to a number of conclusions. First, at pH = 3, the dispersion of the mixtures shows a scattering pattern similar to solutions of individual polymers that did not exhibit significant scattering, as discussed above. In addition, the relatively low scattering intensity for these structures indicates their instability and associative-dissipative nature of existence in an acidic environment with a critically low degree of dissociation of carboxyl groups in the structure of gelatin proteins. Secondly, with an increase to pH = 5.5, the condition pI < pH < pK_0_ is met, and the formation of primary (bio)PEC structures occurs more efficiently, which leads to an increase in the intensity of X-ray scattering due to the presence of a large number of small-sized scattering centers (particles) in the dispersion. Under these conditions, an increase in the proportion of gelatin in the solution additionally leads to a significant increase in the scattering intensity, which is also associated with an increase in the dispersity of the (bio)PEC phase. Moreover, the observed decrease in the amplitude of scattering intensity fluctuations in the curves indicates the formation of more isotropic and stable dispersions. It is also clear that a mixture with a 1:1 ratio at pH = 5.5 has a lower scattering intensity and a noisier signal compared to other mixture ratios at pH = 5.5. We believe that this feature is associated with a strong imbalance in the density of positive and negative charges in the polymer macromolecules with a predominance of positive charges, which leads to a decrease in the efficiency of formation of (bio)PEC structures. This imbalance is due to a significant difference in the molecular masses of biopolymers (about four times) and a difference in the concentrations of functional groups capable of ionization.

**Figure 6:**
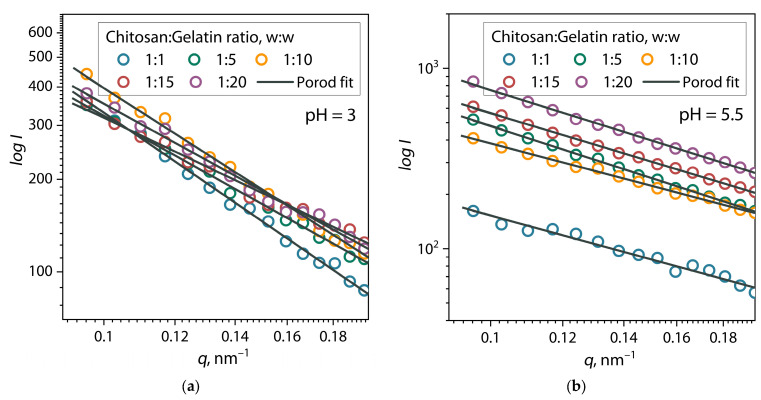
Porod approximation of SAXS patterns of chitosan/gelatin mixtures at (**a**) pH = 3 and (**b**) pH = 5.5.

For a rough analysis of the shape of the primary structures of (bio)PECs, the scattering patterns of chitosan/gelatin mixture dispersions were analyzed in the range *q* < 0.2 nm^−1^ using the Porod approach. Porod plots for (bio)PEC dispersions at pH = 3 and 5.5 are presented in Figure 6.

It can be seen from the figure that these sections of the scattering curves are well described by Equation (2) at *R^2^* > 0.99. The *α* exponent values for these samples according to Equation (2), as well as the fitting parameters of the PDDF function (Equation (3)) and Equations (4) and (5) for a chitosan/gelatin mixtures, are presented in Table 2.

When comparing the *α* exponent values for (bio)PEC dispersions with the values for individual biopolymer solutions (Table 1), it can be observed that they are generally significantly lower. According to the literature, it is known that at a value of the exponent around α ≈ 2, dispersed objects on average have a shape close to a random coil (sphere), and at a value of *α* ≈ 1, close to a rigid rod (one-dimensional rod). According to these calculations, it is clear that the primary structures of (bio)PEC chitosan/gelatin at pH = 3 have a predominantly spherical shape, while at pH = 5.5 they are rod-shaped. However, the shape of the formed structures is not ideal and is intermediate, regardless of the composition of the dispersion. This behavior may be due to some elongation of high molecular weight chitosan chains due to the attachment of small macromolecules of gelatin proteins on its surface during polyelectrolyte interaction and neutralization of the charge of amino acid residues. As a result, it is likely that gelatin macromolecules behave as large substituents along the chitosan backbone and straighten it due to their volume. This concept seems logical given the fact that the energy of electrostatic attraction and binding between polyelectrolytes is higher than the conformation energy of a random chitosan coil in solution, which leads to a shift in the energy equilibrium.

**Figure 7:**
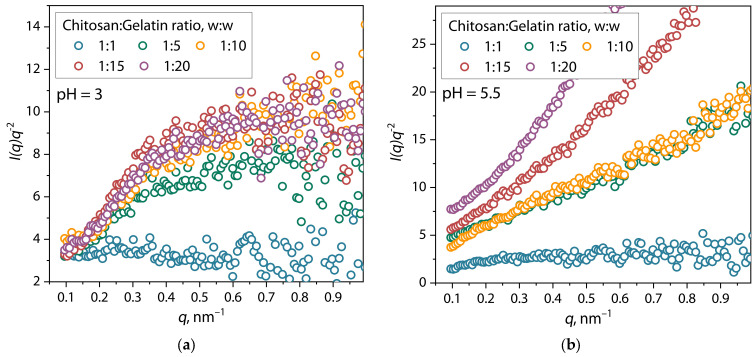
Kratky plots for chitosan/gelatin mixtures at (**a**) pH = 3, (**b**) pH = 5.5.

This behavior is also confirmed by the Kratky plots (Figure 7), which represent the scattering profiles in *I(q)q^2^* vs. *q* coordinates.

**Figure 8:**
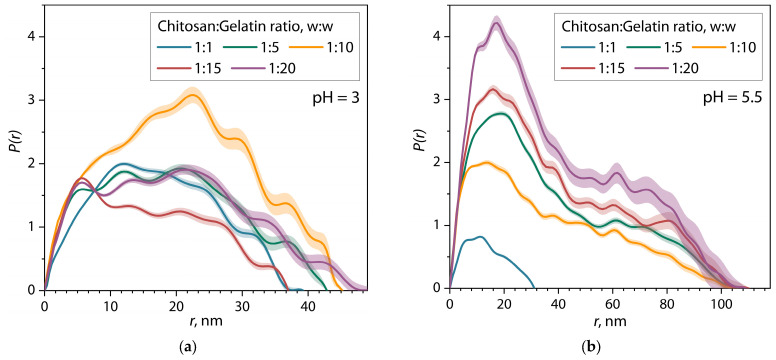
Pair-distance distributions for mixtures of chitosan/gelatin mixtures at (**a**) pH = 3 and (**b**) pH = 5.5.

For mixtures at pH = 3, the Kratky plot initially shows an increase in the *I(q)q^2^* function at low *q* region, followed by a plateau at high *q*, which indicates the presence of structures corresponding to partially unfolded conformations of a random coil [41,46,47]. It is also evident that when the proportion of gelatin in the mixture increases above a ratio of 1:1, the intensity of the function increases significantly, but does not depend on the ratio of polymers in the considered range of *q*. In mixtures at pH = 5.5, the character of the curve changed, showing an increasing slope with increasing fraction of gelatin in the mixture, which indicates the transformation of structural properties from the conformation of a random coil into a fully swollen unfolded spiral [54]. Moreover, the noise of the curve is significantly reduced, indicating the uniformity of the shape of the structural units of the dispersion. For a more accurate analysis of the shape of the structural units of the (bio)PEC dispersion at pH = 3 and 5.5, the analysis of the pair distance distribution function was applied. The corresponding distributions are presented in Figure 8.

The presented distributions for chitosan/gelatin mixtures at pH = 3 (Figure 8a) are monomodal, and their shape is similar to the distributions for individual solutions of biopolymers (Figure 3). Additionally, the *D_max_* and *R_g_* values for the presented distributions practically do not change depending on the ratio of chitosan/gelatin and correspond to the values for the initial biopolymers (Table 1). A different pattern is observed for distributions at pH = 5.5 (Figure 8b). In this case, with ratios above 1:5, bimodal distributions of paired distances are formed, which contain two modes: the first mode up to 50 nm, similar to the size of the initial macromolecules of polymers, and the second mode from 50 to 110 nm (depending on the ratio), which is associated with the formation of elongated objects. Thus, such a form of distribution corresponds to dimeric structures consisting of two domains of macromolecules [38,55]. It is also worth noting that the calculated function parameters show an increase in *D_max_* and *R_g_* by about two to two-and-a-half times compared to the values for mixtures at pH = 3 at ratios above 1:5. Thus, it is evident that the pH value has a significant influence on the size and shape of the formed primary structures of (bio)PEC chitosan/gelatin.

**Figure 9:**
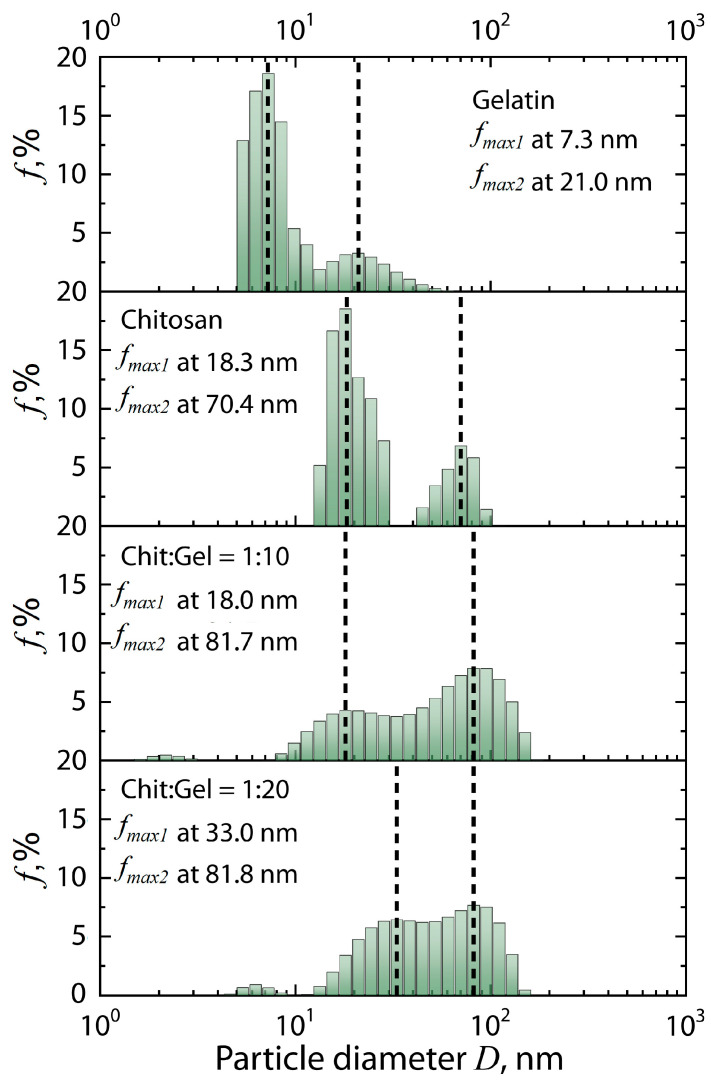
Particle diameter distribution for chitosan and gelatin solutions and their mixtures at pH = 5.5 with different polymer ratios (dashed lines correspond to *f*max).

However, further analyzing the pair distance distribution function, it should be considered that the minimum measured value at *q* = 0.1 nm^−1^ limits the direct determination of particle diameter to about 60 nm. In our case, data analysis using the pair distance distribution function showed that the best fit of the model is observed at *D_max_* = 110 nm. Thus, the *D_max_* value is derived from extrapolation of data and should be interpreted with caution. Therefore, the dynamic light scattering method (DLS) was used to confirm the results obtained by SAXS data. Figure 9 shows the particle diameter distributions for chitosan and gelatin solutions and their mixtures at pH = 5.5, obtained by the DLS method.

It can be seen from the figure that, in the case of individual polymer solutions, bimodal distributions are formed, which are divided into two ensembles consisting of polymer fractions with lower and higher molecular weights, correspondingly. It is worth noting that the comparison of SAXS and DLS data is coupled with some difficulties related to the peculiarities of sample preparation of polymer solutions and their mixtures for DLS. For the DLS method, polymer solutions are usually strongly diluted in order to eliminate the multiple scattering effect, which can distort the experimental results. In our case, polymer solutions and their mixtures were diluted 10 times. On the other hand, for the SAXS method, multiple X-ray scattering is not typical, since the scattering of X-ray photons on atoms is much weaker than the scattering of irradiation in DLS. In addition, in the case of SAXS, the optimal concentration for obtaining a stable signal is more than 1 mg/mL. Therefore, the analyzed solutions for SAXS measurements were not diluted. Thus, the comparison of DLS and SAXS results is qualitative. Despite all of the above, for chitosan and gelatin solutions, we can observe particles comparable in size to those observed according to SAXS and viscometry data. For mixtures of chitosan and gelatin, we observe a simplification of the character of statistical distributions, as well as an increase in the proportion of particles with an average diameter of about 80 nm, compared with solutions of individual polymers. In addition, the most significant result confirming the effective formation of primary structures of (bio)PEC is the presence of particles with an average diameter exceeding the particle diameters in the initial polymers. For example, for mixtures of chitosan and gelatin at pH = 5.5 and a ratio of 1:10 and 1:20, the formation of particles with an average diameter of more than 100 nm can be observed. It is noteworthy that the strong dilution of solutions for DLS measurements also prevented the subsequent interaction of primary particles of (bio)PEC with the formation of coacervates. Therefore, we do not observe large coacervate particles on the diameter distribution, which is a favorable condition for comparing the results obtained by SAXS and DLS. Thus, the DLS results confirm the results of the SAXS data analysis using the pair distance distribution function shown in Figure 8.

**Figure 10:**
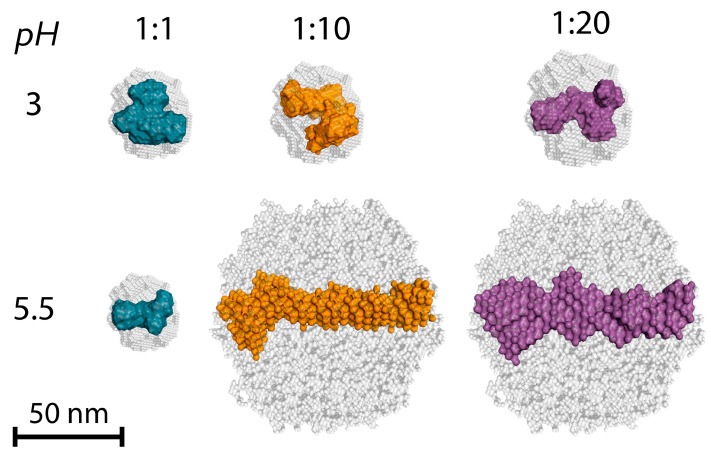
Ab initio models of primary (bio)PEC structures for chitosan/gelatin mixtures with different ratios of polymers at pH = 3 and 5.5.

Further analysis of the shape of structural units in dispersion was carried out using ab initio reconstruction. Figure 10 shows the obtained models of dummy atoms in front projection depending on the ratio of polymers and the pH of the solution.

It can be seen from the figure that the simulation result gives us, as in the case of individual solutions of polymers (Figure 4), a compressed model consisting of tightly packed dummy atoms framed by a sphere (in gray) under initial modeling conditions. The figure shows that the simulation results give particle shapes corresponding to the previously mentioned structures obtained by analyzing with the PDDF function, Kratky plot, and Porod approximation. The particle shape in mixtures of chitosan/gelatin at pH = 3 shows a loose and disordered structure of the oblate spheroid, resembling the shape of the chitosan and gelatin macromolecules. At the same time, the compactness and looseness of the spheroid structure vary regardless of the ratio of polymers. At pH = 5.5, for a ratio of 1:1, a shape similar to that of polymer macromolecules is also observed. However, at higher ratios, there is an increase in longitudinal dimensions in the models. Therefore, elongated structures are formed, the shape of which is close to a cylinder (swollen spiral), which is typical for fully unfolded proteins. Such a form of the structure of primary (bio)PECs is in good agreement with the model proposed by works [14,17], where the formation of metastable elongated aggregates of primary nanoscale PEC based on anionic- and cationic-neutral block copolymers is described in detail.

**Figure 11:**
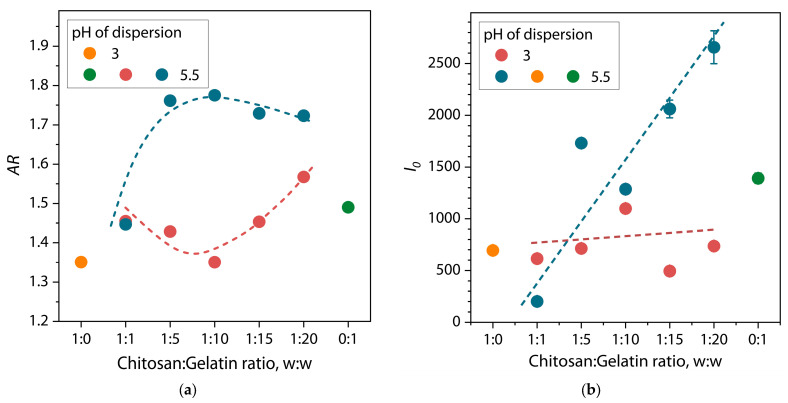
Dependences of (**a**) the aspect ratio *AR* and (**b**) zero scattering intensity *I*_0_ of structural units in dispersions on the chitosan/gelatin ratio with different pH (dashed lines as a guide for the eye).

Additionally, in order to understand how the shape of the analyzed scattering objects changes depending on the pH and composition of the mixture, the aspect ratio *AR* was calculated using Equation (10):

(10) $$AR=\frac{D_{max}}{{2R}_{g}}$$

where *AR* = 1 corresponds to the spherical shape of an object. Figure 11 shows the values of AR for the chitosan–gelatin mixture.

Figure 11 shows the dependences of the aspect ratio of the averaged object in dispersion (*AR* (Equation (10)) and the intensity at zero scattering vector (*I*_0_ (Equation (5)) on the ratio of polymers in the mixture at different pH values.

**Figure 12:**
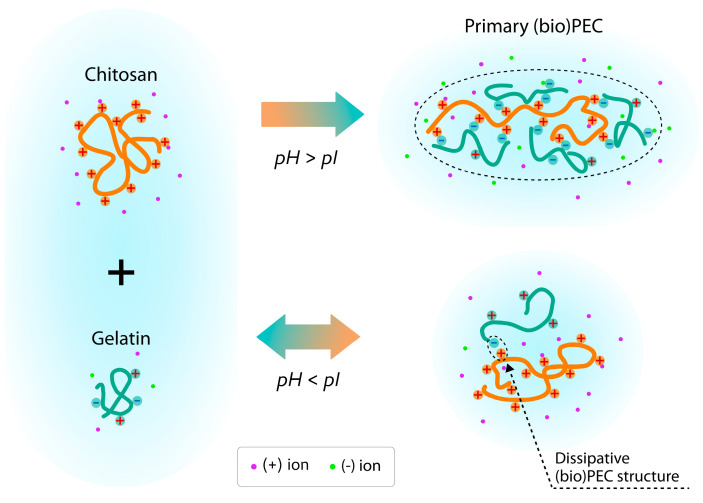
Qualitative scheme of (bio)PEC (borders in dashed circle) formation between chitosan (orange lines) and gelatin (green lines) in solutions with pH below and above pI.

The dependence in Figure 11a shows that at pH = 3, *AR* values fluctuate independently of the ratio of chitosan/gelatin and is close to the values for macromolecules of individual polymers. However, at a ratio above 1:10, *AR* gradually increases, which indicates the slight elongation of the formed average particle. This effect may indirectly confirm the formation of (bio)PEC structures between chitosan and gelatin macromolecules even under acidic conditions, when the dissociation of the acidic groups of gelatin amino acids is suppressed at *pH < pI*. In our previous work [29], this effect was also detected using the dynamic light scattering method on similar dispersions under the same conditions. Here, we believe that under these conditions, structures can be formed consisting of relatively large chitosan macromolecules in the center with one or two gelatin protein macromolecules (based on the values of *D_max_* and *R_g_*) attached by ionic bonds along the main chain. With such an interaction, the main chain of the chitosan macromolecule can straighten somewhat, which can be expressed as an increase in the *AR* value of the average object. A qualitative scheme showing the formation of a (bio)polyelectrolyte complex between chitosan and gelatin can be seen in Figure 12. For mixtures at pH = 5.5, there is a clear dependence on an increase in *AR* value from 1.45 to 1.76, with an increase in the chitosan/gelatin ratio from 1:1 to 1:5, after which it remains unchanged. This result, considering the *pH > pI* conditions of the solution in this case, confirms the proposed structure of (bio)PEC particles and may be a consequence of the increased efficiency of the polyelectrolyte interaction between chitosan and gelatin. Thus, a greater number of gelatin protein macromolecules can be bound to the surface of the chitosan macromolecule at a polymer ratio higher than 1:5 (Figure 12). At the same time, a ratio of 1:1 does not appear due to the insufficient content of negatively charged carboxyl groups for the formation of stable structures with positively charged chitosan amino groups. In this case, as at pH = 3, the process of the formation of (bio)PEC is strongly suppressed.

This conclusion also follows from the dependence in Figure 11b. It is evident that at pH = 3, the *I*_0_ values, which largely depend on the number and size of scattering centers (particles) in the dispersion, change insignificantly for the chitosan/gelatin mixture across the entire range of ratios. However, in the case of pH = 5.5, a sharp and intensive increase in the *I*_0_ values is observed with an increase in the proportion of gelatin in the dispersion. These results also indicate the inefficiency of the polyelectrolyte interaction and the process of formation of (bio)PEC structures at pH = 3, and, conversely, their increased efficiency at pH = 5.5. We believe that at pH = 3, a small number of small (bio)PEC particles are formed, which have low stability under acidic conditions and exist as associative–dissipative structures. As a result, an increase in the proportion of gelatin in the mixture does not significantly affect the number of particles for the scattering intensity. However, at pH = 5.5, the stability of (bio)PEC structures increases significantly, and therefore, with an increase in the proportion of gelatin in the dispersion, their size and quantity can also increase.

## 4. Conclusions

In this work, using SAXS synchrotron radiation, we studied the conformational states of chitosan and gelatin polyelectrolyte macromolecules and the features of their behavior in mixtures at different pH levels and ratios to establish the size and shape of primary supramolecular (bio)PECs. Analysis of the scattering profiles of the initial solutions using the Porod approximation showed that the chitosan and gelatin macromolecules correspond to the 2D conformation of a random coil in a Θ-solvent, with the corresponding q-slope values of 2.26 and 2. The building of the pair distance function of the initial chitosan and gelatin showed the formation of single-modal distributions with maximum molecular sizes, *D_max_*, of 46 and 32.2 nm, respectively. Ab initio reconstruction of the macromolecules’ shape showed the formation of objects shaped like oblate spheroids. In mixtures of chitosan and gelatin at *pH < pI*, it was found that the scattering structures correspond to the initial biopolymers and have a molecular size like them. At the same time, the calculated values of the AR at a ratio above 1:10 gradually increase, which indicates a slight elongation of the average particle. This indirectly indicates the formation of dissipative structures of (bio)PEC even under acidic conditions, when the dissociation of acidic groups of gelatin is suppressed.

However, in mixtures at pH > pI, our results show that at ratios above 1:5, the formation of primary supramolecular complexes is observed, which is accompanied by an increase in *I*_0_ by about three times and *D_max_* by two to two-and-a-half times relative to mixtures below pI. The formation of primary structures of (bio)PEC in mixtures at pH > pI was also confirmed using DLS, where we observed the formation of (bio)PEC particles with a diameter of 100–150 nm. Analysis of PDDF showed that the formation of primary PEC is accompanied by the formation of bimodal distributions with a clearly defined peak of the second mode, which corresponds to elongated dimeric structures consisting of two simple macromolecules of the initial biopolymers. Ab initio reconstruction of the PEC shape showed the presence of elongated structures corresponding to a cylinder (swollen spiral). Also, there is a clear dependence on an increase in *AR* value from 1.45 to 1.76, with an increase in the chitosan/gelatin ratio from 1:1 to 1:5, and then it remains unchanged. This result, considering the pH > pI conditions of the solution in this case, confirms the proposed structure of (bio)PEC particles and may be a consequence of the increased efficiency of the polyelectrolyte associative interaction between chitosan and gelatin.

## Figures and Tables

**Table 1:** Fitting parameters of the pair distance distribution function (PDDF) and the Porod approximation of SAXS patterns of individual chitosan and gelatin solutions at pH = 5.5.

Sample	α	*I*_0_	*D_max_*, nm	Estimate (PDDF)	*R_g_* (PDDF), nm	*R_g_* (Viscometric), nm	*AR*
Gelatin	2.00	1391 ± 50	32.2	0.8332	10.8 ± 0.2	7.5 ± 3.9	1.49
Chitosan	2.26	693 ± 21	46.0	0.6776	17.0 ± 0.1	24.2 ± 4.7	1.35

**Table 2:** Fitting parameters of the pair distance distribution function (PDDF) and the Porod approximation of SAXS patterns for mixtures of chitosan/gelatin at pH = 3 and 5.5.

Chitosan/Gelatin Ratio, *w*/*w*	*α*	*I*_0_	*D_max_*, nm	Estimate (PDDF)	*R_g_* (PDDF), nm
pH = 3					
1:1	2.00	615 ± 17	39.3	0.8751	13.5 ± 0.2
1:5	1.63	713 ± 28	42.8	0.7990	15.0 ± 0.3
1:10	1.87	1099 ± 41	44.0	0.8092	16.3 ± 0.2
1:15	1.39	495 ± 15	37.0	0.7202	12.7 ± 0.2
1:20	1.61	736 ± 38	50.0	0.8609	15.9 ± 0.5
pH = 5.5					
1:1	1.38	201 ± 4	31	0.7988	10.7 ± 0.1
1:5	1.33	1731 ± 38	110	0.7391	31.2 ± 0.7
1:10	1.63	1286 ± 38	108	0.7786	30.4 ± 0.7
1:15	1.52	2061 ± 85	110	0.8683	31.8 ± 1.1
1:20	1.58	2657 ± 159	109	0.8078	31.6 ± 1.6

## Data Availability

The original contributions presented in this study are included in the article. Further inquiries can be directed to the corresponding author.
